# Effects of Dietary Composite Postbiotic Preparation on Growth Performance, Immune Function, and Gut Microbiota in Nubian Black Goats

**DOI:** 10.3390/vetsci13060599

**Published:** 2026-06-20

**Authors:** Yangyan Yin, Changting Li, Yongcui Feng, Huili Bai, Zhe Pei, Zhongwei Chen, Yanwen Zhang, Caifeng Li, Chunxia Ma, Dongyan Deng, Leping Wang, Ling Teng, Hailan Chen, Hao Peng

**Affiliations:** 1 Guangxi Key Laboratory of Animal Breeding, Disease Control and Prevention, College of Animal Science and Technology, Guangxi University, Nanning 530004, China; 2 Guangxi Key Laboratory of Veterinary Biotechnology, Guangxi Veterinary Research Institute, Nanning 530001, China; 3 Key Laboratory of China (Guangxi)-ASEAN Cross-Border Animal Disease Prevention and Control, Ministry of Agriculture and Rural Affairs of China, Nanning 530001, China; 4 Department of Engineering, Virginia Tech, Blacksburg, VA 24601, USA; 5 Office of Research and Academic Development, Nanning University, Nanning 530001, China

**Keywords:** composite postbiotic preparation, Nubian black goat, immune function, growth performance, gut microbiota

## Abstract

This study investigated the effects of composite postbiotic preparation on the growth performance, immune function, and microbiota composition of Nubian black goats. Thirty healthy Nubian black goats with similar body weights were randomly assigned to two groups (n = 15 per group): a control group fed a basal diet and a treatment group fed the basal diet supplemented with 0.5% composite postbiotic preparation (equal-ratio co-fermentation of Bacillus subtilis GX15 and Lentilactobacillus buchneri GX0328-6). The results indicated that while compound postbiotic supplementation did not significantly alter the average daily gain (ADG) and the serum biochemical indices (*p* > 0.05), it significantly increased the concentrations of immunoglobulins (IgG and IgA) compared to the control group (*p* < 0.05). Despite comparable α- and β-diversity, CPP supplementation selectively enriched Bacteroides, UCG-005, and Ruminococcaceae while reducing Turicibacter (LEfSe LDA > 2.0; STAMP *p* < 0.05), suggesting targeted modulation of gut microbiota. In conclusion, dietary supplementation with 0.5% composite postbiotic preparation improves immune function and modulates intestinal microbiota composition without significantly affecting growth performance in black goats while improving intestinal microbial composition and promoting overall gut health.

## Simple Summary

Keeping farm animals healthy is important for agriculture and food production, and farmers are always looking for natural ways to improve animal health without relying too much on antibiotics. This study investigated whether adding a natural ingredient called composite postbiotic preparation to the daily feed of Nubian black goats could improve their overall health. Postbiotics are beneficial compounds produced by microorganisms during the fermentation process. To test this, thirty healthy black goats were divided into two groups: one group was fed a normal diet, and the other group was fed the same diet with a small amount of the postbiotic preparation mixed in. The results showed that although this natural supplement did not make the goats grow faster, it did strengthen their immune systems by increasing the levels of important proteins in the blood that help the body fight disease. The supplement also improved the balance of bacteria living in the goats’ intestines by increasing the number of helpful bacteria and reducing the number of harmful bacteria. In conclusion, adding this natural postbiotic to animal feed is a promising and safe way to boost immunity and improve gut health in goats without using antibiotics. This approach could help farmers reduce drug use and raise healthier animals, which would ultimately provide consumers with safer meat and dairy products while also supporting more environmentally friendly and sustainable farming practices.

## 1. Introduction

Goat breeding is a key sector of specialized animal husbandry, serving as a core source of income and livelihood for small-scale farmers in rural and mountainous regions worldwide [1,2]. Nevertheless, the high cost of conventional commercial compound feeds and high-quality hay has long hindered the sustainable and efficient development of the goat farming industry [1]. This issue has resulted in poor economic benefits and constrained the industry’s further expansion. To address this longstanding constraint, ruminant nutrition and feed researchers have focused on exploring low-cost, locally available feed alternatives and high-nutrient functional additives [3,4]. In arid and rural regions such as Morocco, for example, microalgae, Azolla imbricata (water fern) and Sulla flexuosa (a leguminous herb) have been used as viable alternative feed sources. These natural feed materials are high in crude protein and essential amino acids, alongside valuable bioactive compounds including unsaturated fatty acids, tannins and antioxidants. Such components help optimize rumen fermentation, raise feed digestibility and support goat health, which in turn improves growth performance and the overall quality of goat products [5,6,7]. Numerous studies on indigenous goat breeds in China have likewise confirmed that local plant resources and agricultural by-products can effectively replace conventional feed ingredients. Woody forages such as mulberry leaves and hybrid paper mulberry have proven effective in boosting the growth performance, antioxidant ability and immune status of native breeds including Xiangdong black goats. They also help regulate rumen metabolism and improve the fatty acid profile of goat muscle and milk, as well as enhancing meat quality [8,9]. Moreover, cash crop by-products like ramie leaves can partially substitute for alfalfa hay. When supplemented to the diet of Boer goats and Liuyang black goats, these by-products positively modulate rumen microbial communities, boost nutrient digestibility and improve carcass traits, achieving efficient high-value utilization of agricultural waste [10,11]. Feed additives are widely used in commercial goat farming to improve feed efficiency, promote optimal growth and production performance, and sustain rumen health. Frequently used additives include antimicrobials, enzymes, probiotics, essential oils and plant extracts, all of which contribute greatly to better overall production outcomes in goat rearing [4].

Probiotics are defined as live microorganisms that, when administered in adequate amounts, confer a health benefit on the host [12,13]. In recent years, probiotics have been extensively utilized in livestock and poultry production, with numerous studies documenting their positive effects on disease prevention, immune modulation, and improvement in growth performance [14,15,16,17]. For instance, supplementing the diets of early-weaned goats with lactic acid bacteria has been shown to enhance antioxidant capacity, improve intestinal health, and alleviate weaning-induced diarrhea [18]. Similarly, commercial Narine probiotics (a commercial probiotic product containing Lactobacillus acidophilus) have demonstrated the ability to eliminate Salmonella from the intestinal microbiota of sheep [19]. Furthermore, research indicates that dietary supplementation with Lactobacillus can modulate lipid metabolic pathways in Sunit sheep, thereby influencing intramuscular fat (IMF) deposition and fatty acid (FA) composition, which ultimately improves meat quality and nutritional value [20]. Despite these benefits, the practical application of live probiotics presents certain safety and stability challenges. These include potential risks related to strain pathogenicity, the horizontal transfer of antibiotic resistance genes, and the possibility of triggering allergic reactions, all of which can limit their widespread adoption [21]. To address these concerns, research has increasingly shifted toward postbiotics. Postbiotics are preparations of inanimate microorganisms and/or their components that confer a health benefit on the host. These non-viable preparations consist of inactivated microbial cells, cell fractions (Peptidoglycan, Teichoic Acids, Polysaccharides, and Proteins, etc.), and bioactive metabolites produced during fermentation [12,22,23,24,25]. Beyond their physiological benefits, postbiotics offer superior industrial advantages, including high stability, ease of storage and transportation, and a lower risk profile, representing a promising frontier for probiotic-derived products [13].

The composite postbiotic preparation used in this study were prepared from Bacillus subtilis GX15 and Lentilactobacillus buchneri GX0328-6. Previous studies have confirmed that this combination can alleviate the symptoms caused by Salmonella typhimurium infection in C57BL/6 mice [26,27]. However, when the compound probiotic was fed to cattle and goats for a long time at a ratio of 1:1, no growth-promoting effect was observed, but instead a negative impact on digestion was noted. Given that the efficacy of live probiotics in ruminants is often inconsistent and the underlying mechanisms are complex, the present study turned to a postbiotic preparation strategy, i.e., the use of inactivated microbial cells and their metabolites (such as short-chain fatty acids, bacteriocins, peptidoglycans, etc.), which can play a role in immune regulation and intestinal barrier protection without colonizing the gastrointestinal tract, and offer better environmental stability and processing tolerance [28]. This is the first study of compound postbiotics preparation in Nubian black goats, aiming to evaluate the effects of composite postbiotic preparation (CPP) on growth performance, immune parameters, and intestinal microbiota composition in Nubian black goats.

## 2. Material and Methods

### 2.1. Experimental Materials

The preparation of compound postbiotic preparation (CPP) was as follows. Bacillus subtilis GX15 and Lactobacillus brucei GX0328-6 were activated for two generations, then inoculated into fresh medium at 1% (*v*/*v*). Bacillus subtilis GX15 was cultured in LB liquid medium at 37 °C with shaking at 180 rpm for 24 h. Lactobacillus brucei GX0328-6 was cultured in MRS liquid medium under the same shaking conditions. The cultures containing fermentation supernatant and cells were used directly. The main bioactive components include: lactic acid, raffinose, stachyose, azelaic acid, L-histidine, L-tryptophan, and glycocholic acid, among others. The final bacterial concentration was 109 CFU/mL. The two cultures were mixed at an equal volume ratio of 1:1 (*v*/*v*). The mixture was sterilized by autoclaving at 121 °C for 15 min. Sterility was verified by plate spreading. A total of 100 μL sample was spread on LB and MRS agar plates and incubated at 37 °C for 48 h. No colonies were observed. Commercial assay kits for IgG, IgA, IgM, IL-10, and IL-6 were purchased from Jiangsu Jingmei Technology Co., Ltd.

### 2.2. Experimental Design and Feeding Management

A total of 30 Nubian black goat, all 3 months old and with approximately similar body weights (20.76 ± 1.04), were randomly assigned to two groups using a completely randomized single-factor design. Each group consisted of three replicates, with five goats in each replicate. The control group (Control group) was fed the basal diet, whereas the experimental group (Lb-Bs group) was fed the basal diet (Supplementary Table S1) supplemented with 0.5% CPP. The experimental period lasted for 30 days.

The black goats studied in our research are a black phenotypic strain developed through domestic selection and breeding of the African Nubian goat in China.

### 2.3. Changes in Body Weight of Experimental Nubian Black Goat

The mental status, appetite, and defecation of the experimental black goats were observed daily. On the mornings of the first and last day of the experiment, each goat was weighed before feeding after fasting. Body weight was measured twice consecutively, and the average value was recorded as the initial body weight (IBW) and final body weight (FBW), respectively. The average daily gain (ADG) was then calculated. ADG (Kg/d) = [final body weight (Kg) − initial body weight (Kg)]/30 d [29].

### 2.4. Determination of Serum Immune Indices

At the end of the 30-day experiment, five Nubian black goats were randomly selected from each group. After fasting for 8 h, 5 mL of blood was collected from the jugular vein using non-anticoagulant blood collection tubes. The blood samples were allowed to stand for 30 min and were then centrifuged at 3000 r/min for 20 min at low temperature. The serum was collected and stored at −80 °C. Serum levels of IgG, IgA, IgM, IL-6, and IL-10 were measured using commercially available ELISA kits (Batch No. 202512; Jingmei Biological Technology Co., Ltd., Yancheng, China) according to the manufacturer’s instructions. All samples were assayed in triplicate. Absorbance was read at 450 nm using a multimode microplate reader (TECAN SPARK 10M, Tecan Group Ltd., Männedorf, Switzerland).

### 2.5. Determination of Blood Biochemical Indices

A fully automatic biochemical analyzer was used to determine serum total protein (TP), albumin (ALB), total bilirubin (TBIL), alanine aminotransferase (ALT), alkaline phosphatase (ALP), blood urea nitrogen (BUN), amylase (AMY), glucose (GLU), and cholesterol (CHOL).

### 2.6. Analysis of the Intestinal Microbiota

#### 2.6.1. DNA Extraction

Fresh fecal samples were randomly collected from groups of five Nubian black goats each. Total community genomic DNA extraction was performed using a E.Z.N.A™ Mag Bind Soil DNA Kit (Omega, M5635-02, USA), following the manufacturer’s instructions. We measured the concentration of the DNA using a Qubit 4.0 (Thermo, Waltham, MA, USA) to ensure that adequate amounts of high-quality genomic DNA had been extracted.

#### 2.6.2. 16S rRNA Gene Amplification by PCR

Our target was the V3–V4 hypervariable region of the bacterial 16S rRNA gene. PCR was started immediately after the DNA was extracted. The 16S rRNA V3–V4 amplicon was amplified using 2 × Hieff^®^ Robust PCR Master Mix (Yeasen, 10105ES03, China). Two universal bacterial 16S rRNA gene amplicon PCR primers (PAGE purified) were used: the amplicon PCR forward primer (CCTACGGGNGGCWGCAG) and amplicon PCR reverse primer (GACTACHVGGGTATCTAATCC). The reaction was set up as follows: microbial DNA (10 ng/µL) 2 µL; amplicon PCR forward primer (10 µM) 1 µL; amplicon PCR reverse primer (10 µM) 1 µL; 2 × Hieff^®^ Robust PCR Master Mix (Yeasen, 10105ES03, China) (total 30 µL). The plate was sealed and PCR performed in a thermal instrument (Applied Biosystems 9700, Foster City, CA, USA) using the following program: 1 cycle of denaturing at 95 °C for 3 min, first 5 cycles of denaturing at 95 °C for 30 s, annealing at 45 °C for 30 s, elongation at 72 °C for 30 s, then 20 cycles of denaturing at 95 °C for 30 s, annealing at 55 °C for 30 s, elongation at 72 °C for 30 s and a final extension at 72 °C for 5 min. The PCR products were checked using electrophoresis in 2% (*w*/*v*) agarose gels in TBE buffer (Tris, boric acid, EDTA) stained with ethidium bromide (EB) and visualized under UV light.

#### 2.6.3. 16S Gene Library Construction, Quantification, and Sequencing

We used Hieff NGS™ DNA Selection Beads (Yeasen, 10105ES03, China) to purify the free primers and primer dimer species in the amplicon product. Samples were delivered to Sangon BioTech (Shanghai, China) for library construction using universal Illumina adaptor and index. Before sequencing, the DNA concentration of each PCR product was determined using a Qubit^®^ 4.0 Green double-stranded DNA assay and it was quality controlled using a bioanalyzer (Agilent 2100,Santa Clara, CA, USA). Depending on coverage needs, all libraries can be pooled for one run. The amplicons from each reaction mixture were pooled in equimolar ratios based on their concentration. Sequencing was performed using the Illumina MiSeq system (Illumina MiSeq, San Diego, CA, USA), according to the manufacturer’s instructions.

#### 2.6.4. Sequence Processing, OTU Clustering, Representative Tags Alignment and Biological Classification

After sequencing, The two short Illumina readings were assembled by PEAR software (version 0.9.8) according to the overlap and fastq files were processed to generate individual fasta and qual files, which could then be analyzed by standard methods. The effective tags were clustered into operational taxonomic units (OTUs) of ≥97% similarity using Usearch software (version 11.0.667). Chimeric sequences and singleton OTUs (with only one read) were removed, after which the remaining sequences were sorted into each sample based on the OTUs. The tag sequence with the highest abundance was selected as a representative sequence within each cluster.

#### 2.6.5. Bioinformatics Analysis

Alpha diversity indices (Chao1, Simpson and Shannon) were quantified based on OTU richness. Rarefaction curves of observed OTUs were plotted to assess sampling sufficiency. All alpha diversity indices were calculated using Mothur software (version 3.8.31). OTU rarefaction curves and rank-abundance curve s were drawn in R (version 3.6.0). Beta diversity was used to evaluate microbiome differences between samples. Principal coordinate analysis (PCoA) and Adonis (permutational MANOVA) were conducted and visualized with the vegan package (version 2.5-6) in R. Relative abundances of dominant taxa (≥1%) at phylum and genus levels were calculated. Taxa with abundance below 1% were grouped as “others”. Bar plots of relative abundance were generated in R to compare community structures between the two groups. STAMP (version 2.1.3) and LefSe (version 1.1.0) were used for differential analysis to identify features with significantly different abundances between groups.

### 2.7. Statistical Analysis

Experimental data were analyzed using SPSS 25.0. Normality and homogeneity of variance were tested by Shapiro–Wilk and Levene tests, respectively. To avoid pseudoreplication from group housing, individual values within each pen were averaged to obtain three independent pen replicates for each treatment group. Unpaired independent samples *t*-test was used to compare pen-averaged values between Control and Lb-Bs groups. Differences with *p* < 0.05 were considered statistically significant.

## 3. Results

### 3.1. Changes in Body Weight and Serum Immune Indices of Black Goats Fed the CPP

**Figure 1:**
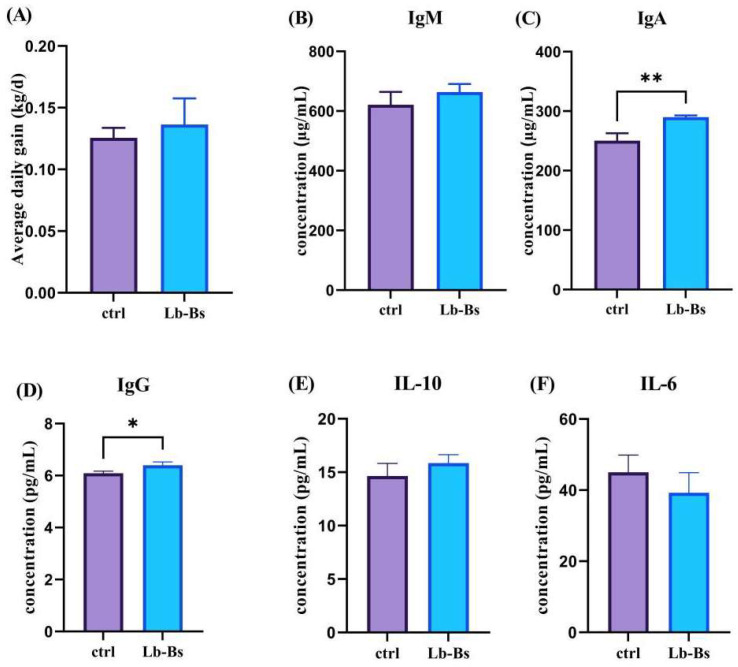
Effects of the CPP on growth performance and serum immune indices in black goats; (∗, *p* < 0.05; ∗∗, *p* < 0.01); (**A**) Average daily gain (ADG); (**B**) IgM; (**C**) IgA; (**D**) IgG; (**E**) IL-10; (**F**) IL-6.

Compared with the control group (Ctrl), there was no significant difference in ADG between the Lb-Bs group and the Ctrl group (*p* = 0.2708; Figure 1A). Serum immune parameter analyses revealed that serum IgA (290.04 ± 2.65 µg/mL vs. 250.34 ± 12.47 µg/mL; *p* = 0.0057; Figure 1C) and IgG (6.39 ± 0.13 g/L vs. 6.09 ± 0.78 g/L; *p* = 0.0276; Figure 1D) concentrations were markedly higher in the Lb-Bs group relative to the Ctrl group. By contrast, no significant intergroup differences were observed for IgM (621.08 ± 43.09 µg/mL vs. 663.59 ± 27.06 µg/mL; *p* = 0.2214; Figure 1B), IL-10 (14.64 ± 1.19 pg/mL vs. 15.85 ± 0.80 pg/mL; *p* = 0.2166; Figure 1E), and IL-6 (45.03 ± 4.82 pg/mL vs. 39.28 ± 5.66 pg/mL; *p* = 0.2514; Figure 1F), even though numerical values of IL-6 were elevated in the Lb-Bs group. Collectively, These findings suggest that the CPP was associated with increased serum IgA and IgG concentrations.

### 3.2. Changes in Serum Biochemical Indices of Black Goats Fed the CPP

As shown in Table 1, compared with the Ctrl group, no significant differences were observed in any of the serum biochemical indices in the Lb-Bs group.

### 3.3. Effects of the CPP on the Intestinal Microbiota of Black Goats

#### 3.3.1. Alpha Diversity Analysis of Intestinal Flora

Alpha diversity analysis showed that there were no significant differences in community richness and diversity indices between the Control and Lb-Bs groups (*p* > 0.05, Table 2). These results indicate that CPP supplementation did not significantly alter the overall richness and diversity of the fecal microbiota in Nubian black goats.

#### 3.3.2. Analysis of Beta Diversity of Intestinal Flora

**Figure 2:**
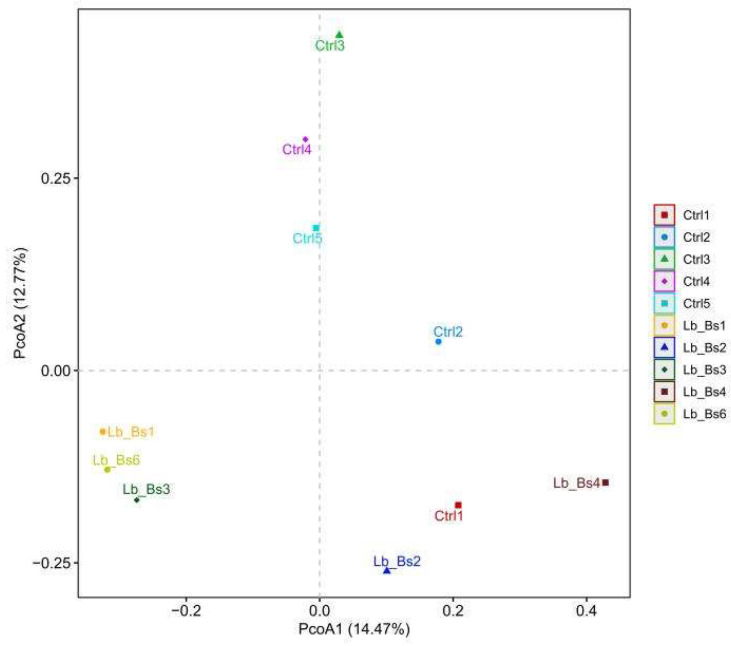
Principal coordinates analysis (PCoA) based on Bray–Curtis distance at the OTU level.

Beta diversity analysis was performed based on the Bray–Curtis distance matrix at the OTU level. Principal coordinates analysis (PCoA) showed that PC1 and PC2 explained 14.47% and 12.77% of the total community variation, respectively (Figure 2). The PCoA plot revealed a slight clustering trend between the Ctrl and Lb-Bs groups. Adonis (PERMANOVA) test results showed F = 1.09, R^2^ = 0.12, and *p* = 0.15, indicating no significant statistical difference in overall microbial community structure between the two groups (Table 3). The grouping factor only explained 12% of the sample microbiota variation.

#### 3.3.3. Sequencing Data and Analysis of Shared OTUs

**Figure 3:**
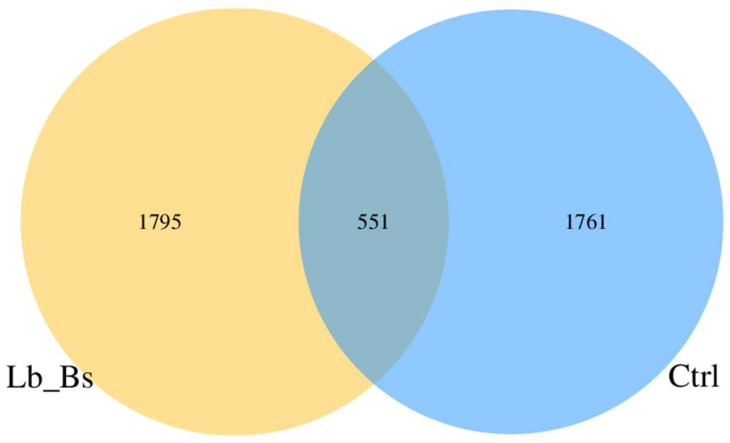
Petal diagram of OTU distribution. Note: The number in the Core represents the OTUs shared among all samples, while the numbers on the petals represent the total OTUs of each sample minus the shared OTUs.

Venn diagram analysis at the OTU level showed that the number of shared OTUs between the Control (Ctrl) and Lb-Bs groups was 551. The Ctrl group had 1761 unique OTUs, while the Lb-Bs group had 1795 unique OTUs (Figure 3).

#### 3.3.4. Differences in the Relative Abundance of Cecal Microbiota at the Phylum and Genus Levels

**Figure 4:**
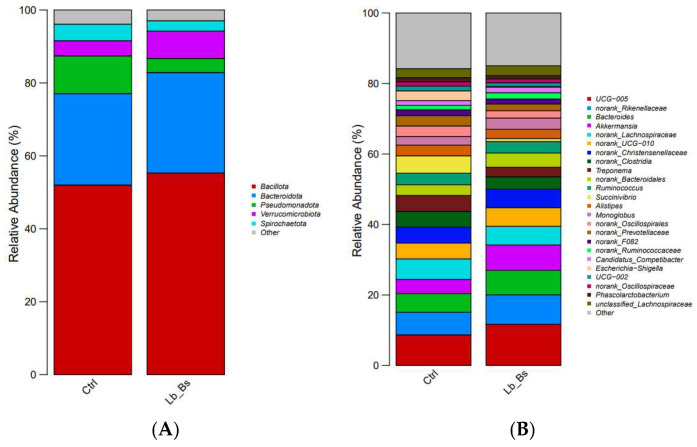
Differences in Relative Abundance at the Phylum and Genus Levels (30 d): (**A**) Phylum level; (**B**) Genus level.

As shown in Figure 4, at the phylum level, the dominant bacterial phyla in each group were *Bacillota*, *Bacteroidota*, and *Pseudomonadota* (Figure 4A). At the genus level, *UCG-005*, unclassified_*Rikenellaceae*, *Bacteroides*, and *Akkermansia* were relatively dominant (Figure 4B).

#### 3.3.5. Differentially Enriched Genera

**Figure 5:**
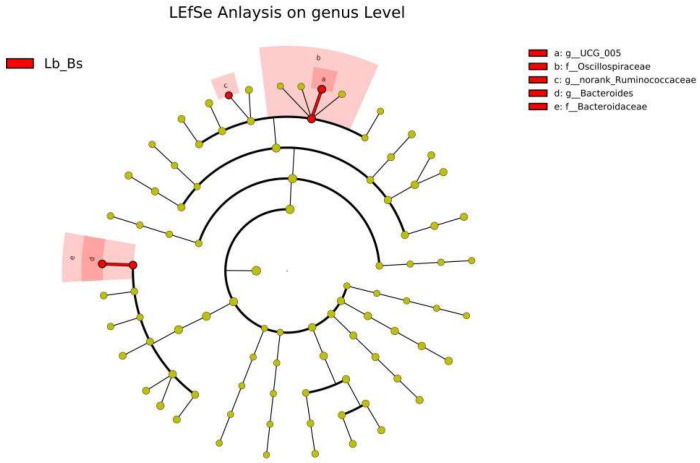
LEfSe cladogram of differentially abundant genera between the two groups. Red branches represent taxa significantly enriched in the Lb-Bs group (LDA > 2.0).

LEfSe analysis (LDA score > 2.0) identified differentially enriched genera between the two groups at the genus level. The cladogram showed that the Lb-Bs group was significantly enriched in *UCG.005* (LDA = 4.23), *Oscillospiraceae* (LDA = 4.18), *Bacteroides* (LDA = 4.08), *Bacteroidaceae* (LDA = 4.08), and g_*norank_Ruminococcaceae* (LDA = 3.91) (red branches). No significantly enriched genera were identified in the Control group. These results suggest that CPP supplementation may exert beneficial effects by selectively promoting the growth of specific bacterial genera (Figure 5).

#### 3.3.6. Analysis of Differential Taxa in the Intestinal Microbiota at the Genus Level

**Figure 6:**
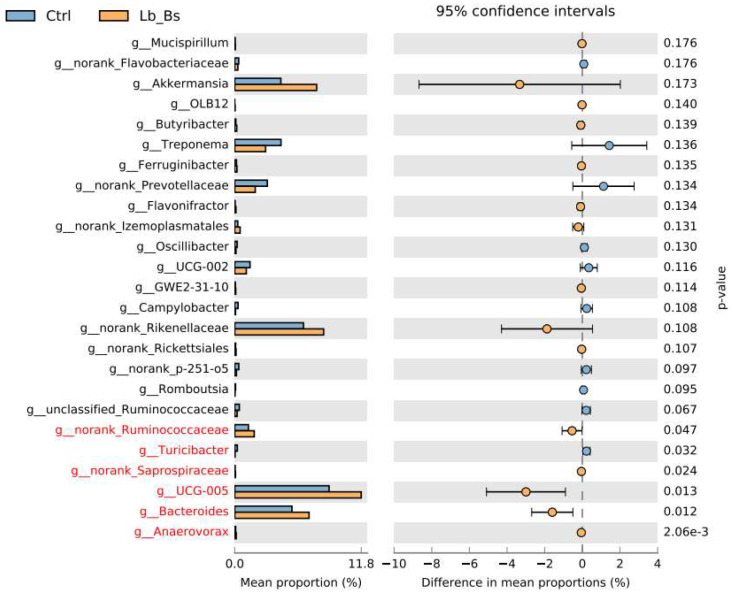
Differentially abundant taxa at the genus level.

As shown in Figure 6, this study investigated changes in microbial relative abundance among different groups through microbial community structure analysis, with a particular focus on bacterial taxa showing significant differences at the genus level. The results indicated that only the genera marked in red showed statistically significant differences between groups. At the genus level, g_norank_*Ruminococcaceae*, g_norank_*Saprospiraceae*, *UCG-005*, *Bacteroides*, and *Anaerovorax* were significantly more abundant in the Lb-Bs group than in the control group, whereas the relative abundance of *Turicibacter* was significantly lower than that in the control group (*p* < 0.05). These findings suggest that these genera exhibited distinct enrichment patterns under supplementation with the compound postbiotic preparation (*p* < 0.05).

## 4. Discussion

In previous studies, a 1:1 composite probiotic of Bacillus subtilis GX15 and Lentilactobacillus buchneri GX0328-6. was used to feed cattle and goats for long-term periods. No growth-promoting effect was observed, but negative effects were produced. The inconsistent performance of live probiotics in ruminants is likely attributable to multiple restrictive factors, rather than a single mechanism. Firstly, the mature rumen of ruminants harbors a stable and highly competitive indigenous microbial community, which generates strong colonization resistance against exogenous microbial strains [30], making it difficult for foreign live bacteria to establish a population advantage in a mature microbial ecosystem. Additionally, the strongly acidic environment (pH 2–3) of the abomasum tends to inactivate most live bacteria [31]. However, it is worth noting that not all probiotic strains require permanent colonization to exert benefits—some can have transient activity and still produce beneficial effects via metabolites or immune signaling [32,33,34]. Postbiotics do not actually contain any live bacteria. Instead, they work indirectly by releasing microbial metabolites—like lactic acid, short-chain fatty acids, and tryptophan derivatives—that influence the gut microbiota, such as certain microbes in the gut consume lactic acid, and in the process, they produce butyrate and other short-chain fatty acids that benefit the host [35,36]. Then there are bile acid hydrolases (BSH) and bile acid-induced enzymes (BAI), which are key players in keeping bile acids balanced and supporting a healthy gut microbiome [37]. Postbiotics can also use adhesion molecules—such as fimbriae [38] and lectins [39]—to compete with other microbes for spots to stick to the gut wall. On top of that, they fight off intestinal pathogens using their metabolites and bacteriocins, stop biofilms from forming, and help deactivate certain harmful microorganisms [40]. Based on this, this study used composite postbiotic preparation prepared from the above strains to intervene, aiming to avoid the technical bottleneck of colonization and survival of live bacteria, and directly play a regulatory role through bioactive substances.

Although many studies have reported beneficial effects of probiotics and postbiotics on animal growth performance and immunity, contradictory findings exist. For instance, some studies found no significant improvement in average daily gain, feed efficiency, or serum biochemical parameters in goats or lambs fed probiotics [41,42,43], and long-term probiotic supplementation even reduced post-weaning growth rates in goats, possibly due to disruption of gastrointestinal microbiota homeostasis in healthy animals [44]. Our results, which showed no significant effect of CPP on growth performance but positive effects on immune parameters and microbiota composition, are partially consistent with these mixed reports. Preliminary observations that the response to postbiotics may be context-dependent and that improvements in immune or microbial endpoints do not necessarily translate into enhanced production performance under good nutritional and management conditions.

The above variations in effects may be closely related to multiple factors, including strain origin, formulation and combination, supplementation dosage, feeding duration, animal breed, rearing environment and other factors [45,46,47].

Probiotics and prebiotics can confer a variety of health benefits by regulating the host immune system, including effects on cellular metabolism, cell proliferation, and intestinal epithelial barrier function [48]. B lymphocytes are the immune cells that produce antibodies, which are antigen-specific immunoglobulins (Igs). This form of protection is called humoral immunity. B lymphocytes also carry Igs on their surface that are capable of binding an antigen. Binding of these surface Igs with antigen causes proliferation of the B lymphocyte and subsequent transformation into plasma cells, which secrete large amounts of antibody with the same specificity as the parent cell [49]. In the present study, black goats fed the compound postbiotic preparation exhibited significantly higher serum IgG and IgA levels than those in the untreated group. This finding is consistent with many previous studies reporting the immune-enhancing effects of probiotics. For example, Ali S.A. Saleem et al. [50]. added a multi-strain probiotic mixture to the basic feed of Saidi male sheep and observed a slight increase in serum IgM levels, and serum IgG and IgA concentrations increased significantly. Zixuan Wang et al. supplemented Ligilaccharissalivarius KS1018 in the basic diet of Xinjiang local breed Duolang sheep to enhance systemic immunity (serum IgG and IgM) and antioxidant status (SOD) [51] In addition, this study found that although supplementation with the compound postbiotic preparation did not significantly alter serum IL-10 and IL-6 levels, it showed a tendency to increase the anti-inflammatory cytokine IL-10 and decrease the pro-inflammatory cytokine IL-6. These results suggest that CPP may enhance the host’s immune response and may have some anti-inflammatory effects; however, since this study did not specifically examine anti-inflammatory properties, future research will systematically investigate this aspect to explore additional functions of CPP.

Serum biochemical parameters reflect nutritional metabolism, hepatic and renal function, and overall metabolic homeostasis. CPP had no significant effects on serum TP, ALB, TBIL, ALT, ALP, BUN, AMY, GLU, or CHOL in black goats. This is consistent with the results of Antunovic et al. [52] and El-Katcha M et al. [53], who reported that feeding diets containing probiotics to growing lambs or goats resulted in no significant differences in total protein, albumin, globulin, and glucose levels. These results suggest that, under the conditions of this experiment, compound postbiotics had no adverse effects on major organ function or basal metabolism in black goats, indicating a high degree of feeding safety. However, Abdel-Salam et al. [54] found that blood concentrations of total protein, albumin, and globulin were greater in lambs receiving probiotic treatments compared to the control diets. These inconsistent findings may be related to multiple factors, including strain origin, host breed, diet composition, and environmental conditions. Based on the observed increases in serum immunoglobulins (IgG and IgA) and alterations in gut microbiota composition in this study, we hypothesize that the postbiotics used here do not directly alter serum metabolic marker levels, but rather indirectly maintain metabolic homeostasis by modulating the intestinal immune microenvironment, activating immune cells, and promoting the secretion of immune factors. However, it must be noted that this hypothesis awaits direct experimental verification. In this study, we did not measure intestinal short-chain fatty acid concentrations, inflammatory cytokine levels (in intestinal tissues), metabolic flux, or digestibility; therefore, we cannot confirm whether the proposed mechanisms actually operate. This represents one of the directions we intend to validate in future studies.

Intestinal dysbiosis can lead to various metabolic, intestinal, and cardiovascular diseases, whereas probiotics have shown beneficial effects as supplements or adjuvant therapies in alleviating these disorders [55]. Maintaining the dynamic balance of intestinal microbiota homeostasis within a stable ecological niche is essential; probiotics can increase the abundance of beneficial bacteria in the gut by promoting the growth of endogenous microbial populations required by the host. A second important mechanism by which probiotics regulate intestinal homeostasis is competitive exclusion, a natural process involving competition for nutrients and ecological niches. This process enhances colonization by beneficial bacteria while preventing the growth of pathogens [56]. No significant differences were found in all alpha diversity indices and beta community structure after postbiotic supplementation (*p* > 0.05). The gut microbial ecosystem maintained stable richness, evenness and overall assembly. Postbiotics did not reshape the whole microbiota but regulated the abundance of specific functional taxa, which matched previous ruminant postbiotic feeding studies [57,58,59]. In this study, we used LEfSe (LDA > 2.0) and STAMP (*p* < 0.05) to find taxa with different abundance. LEfSe found 5 biomarker taxa. STAMP identified 6 significantly different taxa. The two tools gave matching results for g_norank_Ruminococcaceae, UCG-005 and Bacteroides. They disagreed on minor taxa because their calculation methods are different. UCG-005 and Oscillospiraceae were the most abundant taxa in the Lb-Bs group. In the present study, postbiotic supplementation significantly increased the relative abundance of g_norank_Ruminococcaceae, g_norank_Saprospiraceae, UCG-005, Bacteroides, and Anaerovorax in the goat intestine, suggesting that exogenous compound postbiotics can effectively optimize the intestinal microbial community structure and thereby improve intestinal health and nutritional metabolism. The g_norank_Ruminococcaceae, f_Oscillospiraceae and UCG-005 are both core fiber-degrading bacterial groups in the ruminant gut, capable of breaking down structural carbohydrates such as cellulose and hemicellulose to produce short-chain fatty acids (SCFAs) like butyrate [60,61]. Butyrate is not only a major energy source for intestinal epithelial cells but also plays an important role in maintaining the integrity of the intestinal mucosal barrier and alleviating local inflammatory responses [62]. The increased abundance of these bacterial groups in this study leads us to hypothesize that the compound postbiotics may enhance the host’s utilization of crude fiber. Bacteroides is an important commensal genus in the gut that plays key roles in polysaccharide metabolism, maintenance of intestinal homeostasis, and immune regulation. Through four core mechanisms—cholesterol sulfation, bile acid metabolic reprogramming, SCFA-mediated multi-pathway regulation, and anti-inflammatory effects—Bacteroides can systematically reduce cholesterol levels, improve lipid profiles, and lower the risks of atherosclerosis and thrombosis, making it a key microbial target for gut-based interventions [63]. The increased relative abundance of Bacteroides observed in this study leads us to hypothesize a positive regulatory effect of CPP on goat intestinal health. Saprospiraceae has a strong capacity to degrade complex carbon sources [64], while Anaerovorax, which belongs to the order Clostridiales, plays an important role in the degradation of plant fiber in the rumen [65]. Their increased abundance might suggest that compound postbiotic intervention helps maintain intestinal microecological balance and promotes nutrient cycling. However, because we did not measure fermentation metabolites or nutrient flux, this interpretation is speculative. In contrast, Turicibacter showed a significant decrease in the intestines of black goats fed the compound postbiotic preparation. Although the biological characteristics of Turicibacter are not yet fully understood, growing evidence suggests this genus is associated with host disease. For example, the representative strain Turicibacter sanguinis was originally isolated from a patient with acute appendicitis [66]. Additionally, some members of this genus have been reported to act as opportunistic pathogens closely associated with intestinal inflammation [67,68,69,70]. Collectively, these findings indicate that dietary supplementation with compound postbiotics alters the gut microbiota composition in goats, including enrichment of taxa that are inferred from previous literature to be associated with fiber degradation, SCFA synthesis, and homeostasis, as well as reduction in a genus linked to opportunistic pathogenesis. The functional consequences of these compositional changes (e.g., SCFA production, fiber digestibility, cholesterol metabolism, inflammatory status, nutrient cycling) were not directly measured in this study. Therefore, we propose the hypothesis that CPP may improve intestinal health in goats through these pathways, but this hypothesis must be tested in future studies that include direct measurements of relevant metabolites, enzyme activities, and physiological endpoints.

Although probiotics have good growth-promoting and disease-resistant effects in monogastric animals, their application in ruminants is not completely consistent. Based on this, this study turned to the postbiotic intervention strategy, using inactivated cells of probiotic strains and their metabolic products to intervene. This strategy does not rely on living bacterial colonization, does not compete for niche, and can directly play a regulatory role through stable cell wall components and functional metabolites. The results of this study confirmed that the compound postbiotic preparation significantly improved the immune indicators and intestinal flora structure of Nubian black goats, indicating that it is feasible to regulate ruminant nutrition and health through non-viable bacterial pathways, and provides new ideas for the development of ruminant microecological preparations.

## 5. Conclusions

In summary, while research on postbiotics in goat farming remains scarce, this study provides preliminary observational evidence regarding the effects of compound postbiotics (CPP) in Nubian black goats. Specifically, CPP supplementation was associated with modest increases in certain immunoglobulin levels (e.g., IgA, IgG) and alterations in fecal microbial community composition, including shifts in the relative abundances of several genera. However, this study did not demonstrate conclusive improvements in intestinal health, metabolic function, or production performance (e.g., average daily gain, feed efficiency). Importantly, due to the inherent limitations of extrapolating from probiotic-based studies, the unique mechanisms of action of postbiotics remain undefined. Nevertheless, further research—including targeted mechanistic studies and larger-scale trials with direct measurements of intestinal barrier function, metabolic markers, and long-term performance—is required before any definitive conclusions can be drawn regarding the functional benefits of CPP.

## Figures and Tables

**Table 1:** Effects of the CPP on blood biochemical indices of black goats.

Item	Ctrl	Lb-Bs
TP	64.60 ± 1.28 g/L	65.33 ± 0.06 g/L
ALB	36.13 ± 1.47 g/L	35.22 ± 1.81 g/L
TBIL	3.46 ± 0.25 umol/L	4.45 ± 0.32 umol/L
ALT	38.83 ± 0.76 U/L	37.33 ± 6.93 U/L
ALP	398.50 ± 22.50 U/L	316.67 ± 32.53 U/L
BUN	5.17 ± 0.50 mmol/L	5.34 ± 0.41 mmol/L
AMY	172.83 ± 23.61 U/L	205.17 ± 27.90 U/L
GLU	2.97 ± 0.39 mmol/L	2.33 ± 0.47 mmol/L
CHOL	2.62 ± 0.7 mmol/L	2.35 ± 0.18 mmol/L

Note: TP, Total protein (g/L); ALB, Albumin (g/L); TBIL, Total bilirubin (μmol/L); ALT, Alanine aminotransferase (U/L); ALP, Alkaline phosphatase (U/L); BUN, Blood urea nitrogen (mmol/L); AMY, Amylase (U/L); GLU, Glucose (mmol/L); CHOL, Cholesterol (mmol/L). All detected biochemical parameters had *p* > 0.05 between the two groups.

**Table 2:** Comparison of alpha diversity indices between control and Lb-Bs groups.

Sample	Ctrl	Lb-Bs	*p* Value
Shannon	5.6165 ± 0.15563	5.6678 ± 0.12093	0.438
Chao	538.5211 ± 46.91858	549.9247 ± 80.27936	0.339
Simpson	0.0100 ± 0.00722	0.0076 ± 0.00188	0.246

Note: Values are mean ± standard deviation (SD). Statistical significance was determined by independent samples *t*-test.

**Table 3:** Results of Adonis (PERMANOVA) analysis based on Bray–Curtis distance between Ctrl group and Lb-Bs group.

Comparison	F. Model	R^2^	*p* Value
Ctrl vs. Lb-Bs	1.09	0.12	0.15
Between	1.09	0.12	0.14

Note: F.Model: F test statistic; R^2^: Explanatory degree of grouping for community variation; *p* Value: Significance *p* value.

## Data Availability

The original contributions presented in this study are included in the article/Supplementary Material. Further inquiries can be directed to the corresponding authors.

## Supplementary Materials

The following supporting information can be downloaded at: https://www.mdpi.com/article/10.3390/vetsci13060599/s1, Table S1: Ingredients and nutrient levels of the basal diet fed to 3-month-old black goats.
